# Empathy in dental education: a cross-sectional comparative study among undergraduate and postgraduate students in Iran

**DOI:** 10.1186/s12909-026-10090-4

**Published:** 2026-08-07

**Authors:** Mehrnaz Karimi Afshar, Marzieh Karimi Afshar, Hosein Ataollahi

**Affiliations:** 1 Department of Prosthodontics, Faculty of Dentistry, Shiraz University of Medical Sciences, Shiraz, Iran; 2 Department of Orthodontics, Faculty of Dentistry, Kerman University of Medical Sciences, Kerman, Iran; 3 Private practice, Kerman, Iran

**Keywords:** Empathy, Students, Dental, Internship and residency, Education, Dental, Communication

## Abstract

**Background:**

Empathy plays a critical role in effective dentist–patient communication and high-quality care. However, its development during dental education remains inconsistent across studies and contexts.

**Methods:**

A total of 132 dental students and residents (50% male; 22.8% married; 31.8% postgraduate) completed the validated Jefferson Scale of Physician Empathy, assessing perspective taking, compassionate care, and standing in patients’ shoes. Data were analyzed using descriptive statistics, independent t-tests, ANOVA, and linear regression (SPSS 25; *P* < 0.05).

**Results:**

The mean total empathy score was 80.3 ± 7.3 (possible range: 0–140). Female participants scored higher than males in the “Standing in Patients’ Shoes” domain (*P* = 0.025), with no other significant gender differences. No significant differences were observed between undergraduate and postgraduate students in univariate analyses. In multivariate models, marital status was positively associated with total empathy (B = 7.70, *P* = 0.034). Separate regression models examining overall versus domain-specific empathy outcomes demonstrated that “Years of Study” showed inconsistent associations across models, indicating domain-dependent effects that should be interpreted cautiously. Age demonstrated differential associations across domains, with a positive relationship for Perspective Taking (B = 0.62, *P* = 0.021) and negative associations for Compassionate Care (B = − 0.58, *P* = 0.001) and Standing in Patients’ Shoes (B = − 0.20, *P* = 0.015).

**Conclusions:**

Empathy scores varied across domains and were associated with demographic and academic factors in a model-dependent manner. These findings highlight the complexity of empathy in dental education and support the need for longitudinal and context-sensitive educational strategies.

## Introduction

Empathy constitutes a cornerstone of healthcare professionalism, enabling practitioners to understand patients’ emotional and cognitive experiences while fostering trust, treatment adherence, and superior clinical outcomes in dentistry [1–6]. This cognitive attribute—accurately perceiving patient perspectives, communicating understanding compassionately, and committing to supportive action without compromising boundaries—underpins patient-centered care, enhancing diagnostic accuracy, ethical decision-making, and professional satisfaction [7–12].

Although empathy integrates cognitive and affective dimensions, its trajectory during medical and dental training shows variability: international studies report declines due to academic stress, burnout, and workload [13–18], while others document stability or improvements following empathy-focused educational interventions [4, 5].

In Iran, dental student surveys from Tehran and Yazd reveal moderate-to-low empathy levels linked to gender, academic year, hierarchical dynamics, and efficiency-focused curricula, with declines often noted as training advances [19, 20].

To date, Iranian research on empathy in dental education has largely examined undergraduate and postgraduate students separately or compared cohorts across different institutions or time points. Such approaches limit the ability to disentangle the effects of educational stage from institutional, curricular, and cultural influences. Moreover, prior studies have typically focused on a limited set of variables—most commonly gender or academic year—without concurrently evaluating multiple demographic and academic characteristics within a unified analytical framework.

Addressing these limitations, the present study simultaneously assesses and compares empathy levels among undergraduate and postgraduate dental students within a single institution, the School of Dentistry at Kerman University of Medical Sciences. By examining both cohorts at the same time and within the same educational and clinical environment, this design minimizes institutional and temporal confounding and allows for a more precise interpretation of empathy differences attributable to educational stage. In addition, the study evaluates a broader range of demographic and academic predictors—including gender, academic stage, and program duration—providing a more integrated understanding of factors associated with empathy across the dental education continuum in Iran. The present study explores potential differences in empathy levels across demographic and academic factors among undergraduate and postgraduate dental trainees within a unified institutional context.

## Materials and methods

This descriptive cross-sectional study was conducted at the School of Dentistry, Kerman University of Medical Sciences, Iran. The study population comprised all clinical dental students in their fourth to sixth years and all specialty residents currently enrolled at the institution.

### Sample size and sampling method

A census sampling strategy was adopted because the target population at the institution was finite, clearly defined, and fully accessible, and the primary objective was to compare empathy levels across educational stages within the same institutional context rather than to estimate population parameters. Under such conditions, census sampling is methodologically appropriate and minimizes selection bias while maximizing representativeness of the available population. Accordingly, all eligible clinical dental students and specialty residents (*n* = 206) were invited to participate, resulting in 132 completed questionnaires (response rate: 64.07%).

An a priori power calculation was not performed because the study aimed to include the entire accessible population of eligible students and residents at the institution. To enhance transparency, a post-hoc power analysis was additionally reported as a descriptive measure rather than as evidence of sample adequacy. Under assumptions of a medium effect size (d = 0.5), the observed sample corresponded to an estimated statistical power of 0.85 for main comparisons, whereas analyses involving the smaller postgraduate subgroup (*n* = 42) yielded lower estimated power (0.72), indicating a possible increased risk of type II error in subgroup analyses. Comparisons between respondents and non-respondents were limited to available demographic variables (age, gender, and educational level) and showed no statistically significant differences. Nevertheless, the possibility of residual non-response bias related to unmeasured empathy-related characteristics cannot be excluded.

### Procedure

After obtaining ethical approval from Kerman University of Medical Sciences (IR.KMU.REC.1401.043), data collection was coordinated by a trained final-year dental student who was blinded to the study hypotheses. Participants received standardized written and verbal instructions describing the study as an investigation of professional attitudes in dental education and explaining questionnaire procedures, confidentiality, and voluntary participation. Specific hypotheses, constructs, or expected group differences were not disclosed prior to completion to minimize response bias. Following questionnaire completion, a brief debriefing was conducted in which participants were informed about the general research topic (empathy in dental education), the intended academic use of the data, and their right to withdraw their data if desired. No individual scores or comparative results were shared at that stage.

### Data collection instrument

Data were collected using the Jefferson Scale of Physician Empathy–Health Professionals Version (JSE-HP). Two Persian adaptations of the instrument have previously been reported in Iranian populations. Although the Persian adaptation reported by Hashemipour and Karami demonstrated slightly higher internal consistency indices, the version developed by Karimi et al. was selected because it has been more frequently applied in Iranian healthcare student populations and provides wording more closely aligned with contemporary educational terminology used in dental training contexts. This decision was based on prior usage patterns in comparable Iranian student samples rather than superiority of psychometric performance alone. To further support measurement appropriateness in the present sample, internal consistency indices were re-evaluated using the current dataset.

The JSE-HP demonstrated excellent internal consistency in this study (Cronbach’s α = 0.89 for the total scale), with satisfactory reliability for all subscales (Perspective Taking α = 0.82; Compassionate Care α = 0.87; Standing in Patient’s Shoes α = 0.85). The observed subscale reliabilities were comparable to or exceeded those reported in previous Persian validation studies, supporting the appropriateness of domain-level analyses within the present sample.

### Data analysis

Analyses used SPSS v25. Descriptive statistics summarized characteristics and scores. Normality (Shapiro-Wilk) and homogeneity (Levene’s) were confirmed. Categorical variables were analyzed using chi-square tests. Inferential analyses included independent t-tests, one-way ANOVA, Pearson correlation analysis, and multiple linear regression. Regression analyses used a backward elimination procedure in which all candidate predictors were initially entered simultaneously and sequentially removed if *p* > 0.10, with retention at *p* < 0.05. Separate models were constructed for total empathy and for each empathy domain. Multicollinearity was assessed using the Variance Inflation Factor (VIF); all VIF values ranged between 1.12 and 2.31, indicating no problematic collinearity among predictors, including age and years of study. Significance level was set at *P* < 0.05.

### Ethical considerations

The study protocol was approved by the Ethics Committee of Kerman University of Medical Sciences (IR.KMU.REC.1401.043). Participation was voluntary, informed consent was obtained from all participants, and all procedures complied with the Declaration of Helsinki.

## Results

Of 206 eligible students, 132 participated (64% response rate), balanced by gender (50% male/female), predominantly single (77%) undergraduates (68%) (Table 1).

**Table 1:** Frequency distribution of participants by demographic characteristics

Variable	Category	Frequency	Percentage (%)
Gender	Male	66	50.0
	Female	66	50.0
Marital Status	Married	30	22.8
	Single	102	77.2
Program of Study	General (undergraduate)	90	68.2
	Specialty (postgraduate)	42	31.8
Year of study (Specialty)	Fourth Year	5	11.9
	Third Year	10	23.8
	Second Year	14	33.3
	First Year	13	31.0
Year of study (General)	Sixth Year	21	23.3
	Fifth Year	20	22.2
	Fourth Year	21	23.3
	Third Year	28	31.1

### Empathy and domain scores

Mean total empathy score was 80.31 ± 7.34 (out of 140). Domain means: Perspective Taking 38.20 ± 4.98; Compassionate Care 30.73 ± 4.04; Standing in Shoes 11.36 ± 2.14.

### Gender differences

Statistically significant gender differences were observed only in “Standing in Patients’ Shoes” (females 11.79 ± 1.94 vs. males 10.92 ± 2.30; *P* = 0.025, Cohen’s d = 0.41). (Table 2).

**Table 2:** Relationship between gender and empathy and its domains

Variable	Gender		*P* value	Cohen’s d
	Male	Female		
Perspective Taking	37.93 ± 3.76	38.35 ± 6.04	0.701	0.08
Compassionate Care	31.04 ± 3.60	30.41 ± 4.33	0.383	-0.16
Standing in Patient’s Shoes	10.92 ± 2.30	11.79 ± 1.94	0.025*	**0.41**
Total Score	80.01 ± 5.97	80.46 ± 8.45	0.748	0.06

### Program of study

No statistically significant differences were observed between undergraduate and postgraduate students in empathy or its subdomains (all *P* > 0.05; Table 3). Effect size estimates indicated that differences between undergraduate and postgraduate students were small across all domains (Cohen’s d ≤ 0.22), supporting the practical insignificance of the observed non-significant findings.

**Table 3:** Relationship between program of study and empathy and its domains

Variable	Program of Study	Mean ± SD	*P* value	Cohen’s d
Perspective Taking	undergraduate	38.03 ± 4.76	0.757	0.06
	postgraduate	38.34 ± 5.34		
Compassionate Care	undergraduate	30.97 ± 3.80	0.230	-0.22
	Postgraduate	30.06 ± 4.43		
Standing in Patient’s Shoes	undergraduate	11.27 ± 2.00	0.377	0.16
	postgraduate	11.62 ± 2.37		
Total Score	undergraduate	80.27 ± 6.70	0.889	0.03
	Postgraduate	80.07 ± 8.23		

### Predictors of empathy

To examine demographic associations with empathy, correlation and regression analyses were conducted. Age demonstrated a significant negative bivariate correlation with total empathy (*r* = − 0.332, *P* = 0.001). However, domain-specific analyses revealed heterogeneous associations: age was positively associated with Perspective Taking but negatively associated with Compassionate Care and Standing in Patient’s Shoes, indicating that the overall inverse correlation masked distinct cognitive and affective domain patterns. Candidate predictors entered into the regression analyses included age, gender, educational level, marital status, years of study, and academic GPA. Backward elimination regression was subsequently performed using retention criteria of *P* < 0.05 and removal criteria of *P* > 0.10. Academic GPA showed a statistically significant bivariate association with total empathy but was not retained in the final multivariable model after adjustment for age and other covariates (Table 4).

**Table 4:** Correlation and multivariate regression analysis of age and empathy domains

Predictor Variable	*r* (Pearson)	*P* (2-tailed)	β (Standardized)	*P* (Regression)	Model Inclusion
Age	-0.332**	0.001	-0.298**	0.002	Entered
Gender	0.045	0.610	0.071	0.480	Excluded
Year of Study	-0.121	0.190	-0.105	0.210	Excluded
Academic GPA	0.182	0.045	0.153*	0.049	Excluded

Columns 2–3 present bivariate Pearson correlation coefficients between each predictor and total empathy score. Columns 4–5 present standardized β coefficients and associated P values from the initial multivariable regression step prior to backward elimination. Variables retained in the final regression model are identified under “Model Inclusion.” Final retained predictor: Age (β = −0.298, *P* = 0.002). Academic GPA demonstrated a significant bivariate association but was excluded during backward elimination after adjustment for covariates. Regression analyses used backward elimination (retention criterion *P* < 0.05; removal criterion *P* > 0.10). Model multicollinearity was low (all VIF values 1.12–2.31). Final model R² = 0.18, adjusted R² = 0.16, *P* < 0.001

For regression analyses, “Years of Study” was operationalized as a continuous variable reflecting the total number of years spent in formal dental education. For undergraduate students, this corresponded to the academic year of enrollment (years 3–6), while for postgraduate students it represented the number of completed residency years (years 1–4). This harmonized variable F.

Academic GPA was obtained from official institutional records and reflected cumulative academic performance up to the semester preceding data collection. GPA was reported on a 0–20 grading scale, consistent with the national Iranian higher education system.

In domain-specific regression models (Table 5), “Years of Study” emerged as a significant predictor, with higher years associated with lower scores in some domains. However, this variable was not retained in the overall model for total empathy (Table 4), indicating model-dependent effects.

**Table 5:** Multiple linear regression analysis of empathy domains and demographic variables (backward elimination model)

Empathy Domain	Predictor Variable	B (Unstandardized Coefficient)	*P* Value	Model Interpretation
Total Empathy Score	Marital Status	7.70	**0.034**	Being married was associated with a 7.70-point increase in total empathy score
	Years of Study	-1.021	**0.049**	Each additional year of study was associated with a 1.02-point decrease in total empathy score
Perspective Taking	Age	0.621	**0.021**	Each one-year increase in age was associated with a 0.62-point increase in Perspective Taking
	Years of Study	-0.984	**0.039**	Each additional year of study was associated with a 0.98-point decrease in perspective taking
Compassionate Care	Age	-0.580	**0.001**	Each one-year increase in age was associated with a 0.58-point decrease in Compassionate Care
	Marital Status	4.847	**0.003**	Being married was associated with a 4.84-point increase in compassionate care
Standing in Patient’s Shoes	Age	-0.203	**0.015**	Each one-year increase in age was associated with a 0.20-point decrease in Standing in Patient’s Shoes
	Educational Level	2.538	**0.003**	Postgraduate status was associated with a 2.54-point higher score in Standing in Patient’s Shoes

Results are based on separate multiple linear regression models for each empathy outcome using backward elimination regression (retention criterion *P* < 0.05; removal criterion *P* > 0.10). Only predictors retained in the final step are presented. Marital Status was coded as married = 1 and single = 0. Educational Level was coded as postgraduate = 1 and undergraduate = 0. “Years of Study” was treated as a continuous harmonized variable representing progression within the participant’s current educational program. All continuous predictors were mean-centered prior to model entry. The total empathy model shown in this table represents an exploratory supplementary model incorporating demographic variables distinct from those examined in Table 4 and should therefore not be interpreted as directly equivalent to the Table 4 model

The differing role of “Years of Study” between models reflects variation across domain-specific versus overall empathy outcomes and should be interpreted cautiously.

## Discussion

This study assessed empathy among 132 dental students and residents at Kerman University of Medical Sciences using the validated Persian JSE-HP (α = 0.89), revealing a mean total empathy score of 80.31 ± 7.34 within the instrument’s possible range of 0–140. Key findings included females superior in “Standing in Shoes” (11.79 ± 1.94 vs. 10.92 ± 2.30 males; *P* = 0.025, d = 0.41), no undergraduate/postgraduate differences despite simultaneous cohort analysis, age negatively correlated with total empathy (*r*=-0.332, *P* = 0.001), married participants higher overall (β = 7.70, *P* = 0.034), and domain-specific age effects: positive for Perspective Taking (β = 0.621, *P* = 0.021) but negative for Compassionate Care (β=-0.580, *P* = 0.001) and Shoes (β=-0.203, *P* = 0.015) .

Unlike reports of empathy decline during clinical training in several international studies [12, 21–24], the present study did not identify statistically significant overall differences between undergraduate and postgraduate trainees. This pattern aligns with several Iranian studies reporting relatively stable empathy scores across educational stages [17, 19, 20]. However, direct international comparisons should be interpreted cautiously because studies differ substantially in professional discipline, educational systems, cultural contexts, and instrument versions. For example, the South African study by Archer and Turner [25] examined undergraduate medical rather than dental students and used the student version of the Jefferson Scale of Empathy, limiting comparability with the present findings.

The absence of statistically significant program-level differences should not be interpreted as evidence of true equivalence between undergraduate and postgraduate training stages. The present findings may instead reflect limited subgroup statistical power, relative cohort homogeneity within a single institutional environment, and the restricted sensitivity of self-report measures for detecting subtle educational-stage differences. Although shared academic and clinical stressors across training levels may also contribute to these findings, this interpretation remains speculative because stress-related variables were not directly measured in the present study.

Gender findings partially match local trends [26–28] despite mixed international results [21, 29–33]. Married participants’ advantage echoes psychosocial maturity findings [19].

The divergent effects of age across empathy domains highlight the multidimensional structure of empathy and support distinctions between cognitive and affective components. Increased age and clinical exposure were associated with improved Perspective Taking, potentially reflecting maturation of clinical reasoning and professional communication skills. In contrast, older age was associated with lower scores in Compassionate Care and Standing in Patient’s Shoes, findings that may reflect emotional fatigue, desensitization, or adaptation to efficiency-oriented clinical environments described in empathy erosion frameworks. These interpretations should nevertheless remain cautious because workload, burnout, and stress-related variables were not directly measured in the present study. Additionally, although postgraduate students demonstrated slightly lower Compassionate Care scores in univariate analyses (Cohen’s d = − 0.22), this domain-specific directional trend was small and should be interpreted conservatively given the absence of statistically significant overall program differences.

The absence of statistically significant program-level differences should not be interpreted as evidence of true equivalence between undergraduate and postgraduate training stages. The present findings may instead reflect limited subgroup statistical power, relative cohort homogeneity within a single institutional environment, and the restricted sensitivity of self-report measures for detecting subtle educational-stage differences. Although shared academic and clinical stressors across training levels may also contribute to these findings, this interpretation remains speculative because stress-related variables were not directly measured in the present study.

The absence of statistically significant differences between undergraduate and postgraduate students should be interpreted with caution. Rather than indicating true equivalence between educational stages, this finding may reflect several methodological and contextual factors, including limited statistical power for postgraduate subgroup analyses, potential cohort homogeneity within a single institutional setting, and the use of a self-report instrument that may be less sensitive to subtle stage-related differences. While shared academic and clinical stressors across training levels may also contribute, this interpretation remains speculative because stress-related variables were not directly measured in the present study.

Although no significant differences were observed between undergraduate and postgraduate students in univariate analyses, educational level emerged as a significant predictor of “Standing in Patient’s Shoes” in the adjusted model. This suggests the presence of confounding effects, particularly by age and marital status, which may suppress associations in unadjusted comparisons but reveal them after adjustment.

The interpretation of “Years of Study” should be approached with caution, as the harmonization of undergraduate and postgraduate training years assumes equivalence in clinical exposure and responsibility, which may not fully reflect reality.

These findings can be situated within established theoretical models of empathy development and erosion in healthcare education. Empathy erosion theory suggests that sustained exposure to high workload, hierarchical clinical environments, and efficiency-focused curricula disproportionately affects affective empathy, while leaving cognitive empathy relatively preserved. Simultaneously, professional identity formation models emphasize that learners internalize clinical norms and values over time, which may strengthen perspective-taking abilities while constraining emotional engagement. The observed pattern in this study aligns with the intersection of these frameworks and underscores the role of curricular and institutional context in shaping empathy trajectories.

### Implications for practice

The present findings suggest that empathy training in dental education should explicitly distinguish between its cognitive component (Perspective Taking) and affective components (Compassionate Care and Standing in Patients’ Shoes), as these dimensions may follow different developmental trajectories during clinical training. Particular attention should be given to strengthening affective empathy during the mid-to-late stages of clinical education, when exposure to clinical workload and emotional demands may increase vulnerability to its decline.

From a pedagogical perspective, reflective writing, guided patient narratives, and structured debriefing following emotionally challenging clinical encounters may represent effective strategies for sustaining empathic engagement within routine training environments. In addition, incorporating gender- and age-sensitive mentorship, alongside resilience-building approaches, may help address heterogeneity in empathy development across learner profiles.

More broadly, these findings support the integration of empathy development as a longitudinal, system-level component of clinical education rather than as isolated workshops, thereby aligning affective and cognitive skill development with real-world clinical practice.

### Limitations

- Cross-sectional design precludes causal inference or assessment of longitudinal trajectories.
- Self-reported empathy may be subject to social desirability bias, potentially inflating scores; behavioral validation is warranted.
- Single-institution sample limits generalizability.
- 64% response rate may introduce selection bias toward more empathic participants.
- Unmeasured confounders, such as personality traits, prior clinical exposure, or mental health, could influence results.
- Harmonization of “Years of Study” across undergraduate and postgraduate programs assumes equivalence in clinical exposure, which may not fully reflect reality.

## Conclusion

This study highlights domain-specific patterns of empathy among Iranian dental students and residents, with cognitive aspects preserved and affective components more vulnerable to decline. Age, marital status, and gender demonstrated differential associations across empathy domains. These findings underscore the importance of targeted, theory-informed educational interventions that prioritize sustaining affective empathy while reinforcing cognitive perspective-taking skills. Curricular strategies should be context-sensitive and longitudinally integrated, rather than uniform, to effectively support the development of holistic empathic competencies.

## Data Availability

The datasets used and/or analysed during the current study are available from the corresponding author on reasonable request.
